# The Role of Dipeptide Repeats in C9ORF72-Related ALS-FTD

**DOI:** 10.3389/fnmol.2017.00035

**Published:** 2017-02-13

**Authors:** Brian D. Freibaum, J. Paul Taylor

**Affiliations:** ^1^Department of Cell and Molecular Biology, St. Jude Children’s Research HospitalMemphis, TN, USA; ^2^Howard Hughes Medical InstituteChevy Chase, MD, USA

**Keywords:** amyotrophic lateral sclerosis, C9orf72, dipeptide repeat, frontotemporal dementia, liquid–liquid phase separation, nuclear pore, nucleocytoplasmic transport, RNA metabolism

## Abstract

Expansion of a hexanucleotide (GGGGCC) repeat in the gene chromosome 9 open reading frame 72 (*C9ORF72*) is the most common cause of amyotrophic lateral sclerosis and frontotemporal dementia (FTD). Three non-exclusive mechanisms have been proposed to contribute to the pathology initiated by this genetic insult. First, it was suggested that decreased expression of the C9orf72 protein product may contribute to disease. Second, the recognition that *C9ORF72*-related disease is associated with accumulation of GGGGCC repeat-containing RNA in nuclear foci led to the suggestion that toxic gain of RNA function, perhaps related to sequestration of RNA-binding proteins, might be an important driver of disease. Third, it was subsequently appreciated that GGGGCC repeat-containing RNA undergoes unconventional translation to produce unnatural dipeptide repeat (DPR) proteins that accumulate in patient brain early in disease. DPRs translated from all six reading frames in either the sense or antisense direction of the hexanucleotide repeat result in the expression of five DPRs: glycine–alanine (GA), glycine–arginine (GR), proline–alanine (PA), proline–arginine (PR) and glycine–proline (GP; GP is generated from both the sense and antisense reading frames). However, the relative contribution of each DPR to disease pathogenesis remains unclear. Here, we review evidence for the contribution of each specific DPR to pathogenesis and examine the probable mechanisms through which these DPRs induce neurodegeneration. We also consider the association of the toxic DPRs with impaired RNA metabolism and alterations to the liquid-like state of non-membrane-bound organelles.

## Introduction

The seminal discovery of a GGGGCC hexanucleotide repeat expansion within chromosome 9 open reading frame 72 (*C9ORF72*) in 2011 represented a major advance in neurodegenerative disease research by revealing the most common genetic insult responsible for the development of amyotrophic lateral sclerosis (ALS) and/or frontotemporal dementia (FTD) (DeJesus-Hernandez et al., 2011; Renton et al., 2011; Majounie et al., 2012). The expansion occurs within the noncoding region between exons 1a and 1b of *C9ORF72*; therefore, the expansion occurs in either the first intron or the promoter region of the gene, depending on which of the two alternative transcription start sites is used (DeJesus-Hernandez et al., 2011). Healthy individuals have been described as having up to 23 hexanucleotide repeats, although most carry between two and eight repeats (DeJesus-Hernandez et al., 2011; Renton et al., 2011). Patients with C9orf72-mediated ALS/FTD have greater than 30 hexanucleotide repeats, but repeats that are thousands of base pairs long are common.

Two distinct, though non-exclusive, hypotheses initially described the mechanisms by which this expansion causes disease: (1) loss of function due to decreased expression of the C9orf72 protein; and (2) gain of function due to the accumulation of toxic RNA foci (Taylor et al., 2016). Supporting the loss-of-function hypothesis was the observation that the hexanucleotide repeat expansion leads to modestly decreased protein expression of C9orf72 in both cell culture and affected tissues (DeJesus-Hernandez et al., 2011; Renton et al., 2011; Gijselinck et al., 2012; Belzil et al., 2013). More recent evidence has emerged from studies of *C9orf72*-null mice, which display an age-related inflammatory response in macrophages and microglia, suggesting that loss of C9orf72 protein could potentially contribute in a non-cell autonomous manner to the neurodegeneration observed in patients with ALS/FTD (O’Rourke et al., 2016). However, selective removal of *C9orf72* from neurons does not lead to neurodegeneration or motor defects, suggesting that loss of protein function plays an auxiliary role in the pathogenesis of ALS/FTD (Koppers et al., 2015; O’Rourke et al., 2016).

**Figure 1:**
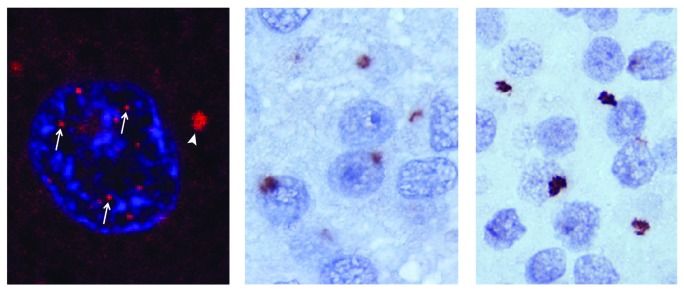
**RNA and DPR pathology in C9orf72 mediated ALS/FTD.** RNA foci in the nucleus (arrows) and the cytoplasm (arrowhead) of a chromosome 9 open reading frame 72 (C9orf72) amyotrophic lateral sclerosis frontotemporal dementia (ALS/FTD) cortical neuron (left). Glycine-alanine (GA; middle) and glycine-arginine (GR; right) dipeptide repeat (DPR) pathology in the dentate nucleus of a C9orf72 ALS/FTD affected brain. Adapated from Taylor et al. (2016).

The accumulation of RNA foci (Figure 1) found predominantly in the nuclei of affected cells was observed concomitant with the discovery of the expanded GGGGCC repeat (DeJesus-Hernandez et al., 2011). Shortly thereafter, it was observed that bidirectional transcription of the expanded repeat leads to the formation of antisense RNA foci (Gendron et al., 2013; Mizielinska et al., 2013). More detailed *in vitro* studies revealed that the GGGGCC repeat adopts a G-quadruplex structure (i.e., a tertiary structure formed by guanine-tetrad stacking), although it remains unclear whether the G-quadruplex is the predominant structure found in sense or antisense RNA foci *in vivo* (Fratta et al., 2012; Reddy et al., 2013). Linking these RNA foci to toxicity, the prevailing gain-of-function hypothesis posits that RNA foci sequester RNA-binding proteins, leading to disruptions in RNA metabolism. Indeed, antisense oligonucleotides directed against the sense strand of the repeat mitigate pathology in an induced pluripotent stem cell model of disease (Donnelly et al., 2013).

The third and most recent non-exclusive hypothesis linking the C9orf72 expansion to pathogenesis is based on the phenomenon of repeat-associated non-ATG (RAN) translation, a type of unconventional translation first observed in the microsatellite expansion disease spinocerebellar ataxia type 8 (Zu et al., 2011). This observation led to interrogation of potential RAN translation of *C9orf72*, which was confirmed by the observation of dipeptide repeats (DPRs) translated from both the sense and antisense strands of the expanded hexanucleotide repeats. From the six reading frames that undergo RAN translation, five distinct DPRs are generated: glycine-alanine (GA) and glycine-arginine (GR) DPRs from the sense RNA strand; proline-alanine (PA) and proline-arginine (PR) from the antisense strand; and glycine-proline (GP) translated from both the sense and antisense strands (Mori et al., 2013a,c; Zu et al., 2013).

The relative pathogenic contributions of loss of C9orf72 protein expression, RNA foci-mediated toxicity, and RAN-generated DPR toxicity remain obscure. However, strong evidence has demonstrated that DPRs are toxic in both cell culture and animal models of disease. However, the relative contribution of each DPR to pathogenesis remains disputed. In this review article, we summarize the current knowledge of the individual contribution of specific DPRs to disease progression and provide a framework for understanding the potential mechanisms by which these DPRs produce their toxic phenotypes.

## Dipeptide Repeat Pathology

A unique pathologic hallmark of C9orf72-mediated disease is the presence of star-shaped cytoplasmic inclusions in both neurons and glia (Figure 1) (Ash et al., 2013; Mann et al., 2013; Mori et al., 2013a,c; Schludi et al., 2015). These inclusions are negative for TDP-43 but positive for p62, ubiquitin, and the five DPR species generated by RAN translation of the hexanucleotide repeat; however, patients also display prominent TDP-43 pathology in degenerating regions of the brain and spinal cord. DPR inclusions have been identified in the hippocampus, basal ganglia, frontal cortex, cerebellum and motor cortex in patients with ALS/FTD (Al-Sarraj et al., 2011; Cooper-Knock et al., 2012; Hsiung et al., 2012; Mahoney et al., 2012; Troakes et al., 2012). The inclusions are also present, albeit in reduced quantities, in the spinal cord (Gomez-Deza et al., 2015; Schipper et al., 2016). DPR pathology in addition to RNA foci have also been observed in transgenic mouse models of C9orf72-mediated disease with varying degrees of associated neurodegeneration (O’Rourke et al., 2015; Peters et al., 2015; Jiang et al., 2016; Liu et al., 2016).

Although the large p62-positive cytoplasmic aggregates are the most prominent feature in the frontal cortex of patients with C9orf72-mediated disease, intranuclear aggregates have also been described. Studies of post-mortem tissue have reported p62-negative para-nucleolar aggregates (Schludi et al., 2015), as well as PR DPR localization in nucleophosmin-positive intranuclear inclusions (Wen et al., 2014), suggesting that nucleolar localization of DPR-containing aggregates occurs in C9orf72-mediated disease. Importantly, multiple reports indicate that DPR pathology appears early in disease, perhaps years before the accrual of TDP-43 pathology (Baborie et al., 2015; Vatsavayai et al., 2016). In some instances a lack of correlation has been described between the regional burden of DPR proteins and the corresponding severity of neurodegeneration, raising questions about their relative contribution to disease (Mackenzie et al., 2013; Davidson et al., 2014). However, these studies were based on the post-mortem examination of brains with end-stage disease and relied on the detection of large inclusions using immunohistochemistry, an approach that likely under-represents the pathological burden of soluble DPR proteins. Moreover, it may be erroneous to assume that insoluble DPRs that accumulate in cytoplasmic aggregates represent the most toxic species, since some DPRs are charged and highly soluble and may be difficult to assess using traditional immunohistochemistry in fixed, post-mortem tissue. Furthermore, it may be difficult to visualize the most toxic species in patient tissues, as degenerating neurons are lost. Nevertheless, the apparent discrepancy between the burden of DPR-protein deposition, the levels of which are greatest in the cerebellum, and the severity of neurodegeneration, which is greatest in the motor cortex and spinal cord, needs to be resolved to understand the role of DPR proteins in the development of disease.

## GA Toxicity

**Figure 2:**
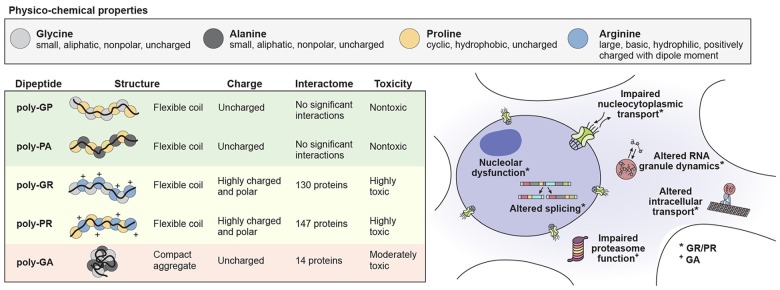
**The physico-chemical properties of individual DPR species influence their toxicity leading to cellular impairment.** Physico-chemical properties of amino acids comprising the DPR species in C9orf72 mediated ALS/FTD (top). The structure and charge of individual DPR species and their influence on their interactions and toxicity (bottom left). Cellular functions known to be impaired by DPR toxicity (bottom right).

Of the five DPR species generated in C9orf72-mediated disease, the GA DPR is the most readily visible in p62/ubiquitin positive inclusions in the brain and spinal cord of patients with ALS/FTD (May et al., 2014; Zhang et al., 2014). Consistent with the expected biophysical properties of a polymer formed from poly-GA (see Figure 2), the GA DPR has a strong propensity to aggregate, forming amyloidogenic fibrils that stain positive with Congo red or thioflavin T (May et al., 2014; Chang et al., 2016). These fibrils form a parallel β-sheet structure that is hypothesized to share similar properties with the structure of the amyloid-beta peptide present in Alzheimer’s disease (Chang et al., 2016; Edbauer and Haass, 2016).

Expression of the GA DPR in neuronal cell cultures leads to reduced dendritic branching, increased endoplasmic reticulum stress, proteasomal inhibition and apoptosis through the activation of caspase 3 (May et al., 2014; Zhang et al., 2014). The GA DPR has been proposed to induce such cellular toxicity through direct sequestration of proteins, with direct proteasomal impairment and interaction with Unc119 as possible primary toxic mechanisms. Supporting this hypothesis, mass spectrometry analysis revealed that, in addition to p62, the GA DPR binds Unc119, ubiquilin-1 and -2, and some proteasomal subunits (May et al., 2014; Schludi et al., 2015). The GA DPR was found to directly associate with Unc119, a protein that regulates axonal maintenance and suppresses axonal branching (Knobel et al., 2001; May et al., 2014). Overexpression of Unc119 in cortical neurons mitigates toxicity arising due to expression of the GA DPR whereas knockdown of Unc119 was sufficient to induce toxicity in the absence of DPR expression (May et al., 2014). A recently developed mouse model showed consistent results by expressing high levels of GA DPR in cortical tissue by adeno-associated viral transduction. These mice showed motor and cognitive deficits along with cerebellar atrophy, neural toxicity and astrogliosis (Chew et al., 2015; Zhang et al., 2016). Consistent with the toxicity observed in cell cultures, the mice showed sequestration of the proteasome proteins HR23A and HR23B by the GA DPR. Overexpression of HR23B partially mitigates poly-GA-induced toxicity, supporting the contention that sequestration of targets by the GA DPR is a driving force behind the associated neurodegeneration.

Although there is general agreement that the GA DPR is toxic when expressed at high levels, the GA DPR appears to be less toxic than arginine-containing DPRs (GR and PR) when compared head-to-head in a common biological system (Mizielinska et al., 2014; Wen et al., 2014; Freibaum et al., 2015). Indeed, multiple studies reported little or no toxicity caused by GA DPR expression (or PA and GP DPR expression for that matter) in primary neurons or *Drosophila* models, but quite potent toxicity of GR and PR DPR expression. Expression of the GA DPR in these model systems is likely to be much lower than in viral-mediated expression systems and may account for the differences in observed toxicity. It remains to be determined whether the GA DPR is toxic at physiologically relevant concentrations and conditions.

## GR/PR Toxicity

Studies demonstrating the toxicities of GR and PR DPRs *in vitro* and in *Drosophila* provided initial insight into the toxicity of these DPRs in C9orf72-mediated ALS/FTD. These DPRs are both highly charged and polar (Figure 2), tend to accumulate in the nucleoli of U2OS cells and cause cell death when added directly to cell culture media at concentrations of less than 10 μM (Kwon et al., 2014). Notably, the stabilities of these two DPRs are quite different from one another: the GR DPR is unstable under cell culture conditions (half-life < 30 min), whereas the PR DPR is relatively stable (half-life ~72 h; Kwon et al., 2014). These DPRs also consistently show toxicity when expressed by plasmid transfection in several different cell lines, inducing cell death in HEK293T cells, NSC-34 cells, as well as both cortical and motor neurons derived from induced pluripotent stem cells (Wen et al., 2014; Tao et al., 2015; Lee et al., 2016).

In *Drosophila*, tissue-specific expression of GR and PR via traditional ATG-mediated translation (using alternative codons to GGGGCC) is extremely toxic in the eye, neuronal tissue (neurons and glia) and motor neurons (Mizielinska et al., 2014; Wen et al., 2014; Freibaum et al., 2015; Lee et al., 2016). In contrast, expression of GA, PA and GP DPRs is not toxic in the *Drosophila* eye. Expression of PA and GP DPRs in *Drosophila* neurons is nontoxic, and expression of the GA DPR leads to only a slight decrease in lifespan (Mizielinska et al., 2014; Wen et al., 2014; Freibaum et al., 2015; Lee et al., 2016). Together, these findings suggest that GR and PR, but not the other DPRs, are highly detrimental to the survival of neurons in *Drosophila*.

Notably, two additional *Drosophila* models of C9orf72 disease confirmed expression of the GR DPR via RAN translation of a GGGGCC repeat (Mizielinska et al., 2014; Freibaum et al., 2015). Insertion of periodic stop codons between the hexanucleotide repeats eliminated the production of RAN-generated DPRs and associated toxic phenotypes in *Drosophila* while retaining a G-quadruplex RNA structure (Mizielinska et al., 2014). One interpretation of this finding is that RNA-mediated toxicity is nonexistent or irrelevant; however, this may be an over-interpretation because it has not been determined whether this modified RNA sequence can sequester the same proteins as can the GGGGCC repeat sequence.

The mechanisms by which GR and PR DPRs induce toxic phenotypes in neurons have become increasingly understood in recent years. Recent publications demonstrated that GR and PR DPRs play a broader role in influencing the formation of membrane-less organelles and their liquid–liquid phase separation dynamics (Lee et al., 2016; Lin et al., 2016). In these studies, initial clues were found by proteomic analysis of the binding partners of GR and PR DPRs, which revealed enrichment in proteins that contain a low complexity sequence domain (LCD) (Lee et al., 2016; Lin et al., 2016). LCDs are evolutionarily conserved amino acid segments, typically 75–300 amino acids in length and present in up to one-third of the human proteome, that are compositionally biased in amino acid representation (Brangwynne et al., 2015). Most often, LCDs are highly enriched in glycine and serine, with interspersed aromatic and charged residues, and are predicted to be unstructured (Huntley and Golding, 2002; Uversky, 2002). LCDs engage in multivalent, low affinity interactions and over the past few years it has emerged that LCD interactions are important for innumerable cellular processes, including the formation and function of membrane-less organelles such as the nucleolus, nuclear pore complex, stress granules, nuclear speckles and Cajal bodies (Brangwynne et al., 2015). These DPRs were found to associate with low-complexity domains exclusively in a polymeric conformation suggesting that the polymeric structure of these DPRs is the primary toxic species *in vivo* (Lin et al., 2016). To assess the contribution of LCD interacting proteins in DPR-mediated toxicity, a *Drosophila* model in which expression of a GR DPR results in decreased viability was utilized (Lee et al., 2016). Using this model for an *in vivo* screen, they used RNA interference to reduce expression of *Drosophila* orthologs of the identified GR- and PR-interacting proteins. Results from this screen demonstrated that reduction of these interacting proteins either enhanced (21%) or suppressed (63%) the viability phenotype caused by expression of the GR DPR.

The most obvious visual association of the GR and PR DPRs with membrane-less organelles is their nucleolar localization in cells (Kwon et al., 2014; Wen et al., 2014; Tao et al., 2015; Lee et al., 2016). PR DPRs are almost exclusively localized within the nucleolus, whereas GR DPRs are observed both in the nucleolus and within the cytoplasm (Wen et al., 2014; Lee et al., 2016). This localization to the nucleolus is not benign, instead resulting in impaired rRNA synthesis, ribosome biogenesis and translation (Tao et al., 2015; Kanekura et al., 2016; Lee et al., 2016). Recent super-resolution fluorescent imaging of the ultrastructure of the nucleolus revealed that both the GR and PR DPRs localize to the granular component, the most distal region of the nucleolus where ribosome assembly takes place (Boisvert et al., 2007; Lee et al., 2016). *In vitro* co-incubation of the GR or PR DPR with nucleophosmin, a protein found in the granular component of nucleoli, induced a liquid–liquid phase separation of nucleophosmin and impaired its interaction with SURF6, an endogenous binding protein (Lee et al., 2016). In live cells, expression of the GR and PR DPRs reduced the mobile fractions of proteins that associate with numerous membrane-less organelles including the nucleolus, stress granules, nuclear speckles and Cajal bodies.

The alteration of liquid-like properties in nuclear speckles by GR and PR DPRs provides a potential mechanism by which these DPRs induce specific splicing alterations in cells (Kwon et al., 2014; Kanekura et al., 2016). GR and PR DPRs also associate with TDP-43-positive RNA granules or stress granules in live cells. Addition of these DPRs causes the granules to become poorly dynamic, which may drive the fibrillization of ALS-causing proteins such as hnRNPA2B1 or TDP-43 (Molliex et al., 2015). Impairment of RNA-granule dynamics by GR and PR DPRs may also affect the initiation stage of RNA granule formation, as they appear to prevent stress granule formation, at least in response to arsenite-induced stress (Tao et al., 2015).

Liquid–liquid phase separation is thought to be the mechanism by which cargoes are selectively transported through the nuclear pore (Schmidt and Görlich, 2016). Recently, several studies have demonstrated that nucleocytoplasmic transport abnormalities are common in *Drosophila* and induced pluripotent stem cell models of C9orf72-mediated ALS/FTD (Donnelly et al., 2013; Freibaum et al., 2015; Jovičić et al., 2015; Zhang et al., 2015). Subsequent *Drosophila* and yeast phenotypic screens have shown that numerous nuclear pore components and proteins involved in shuttling either suppress or enhance the toxicity caused by GR and PR DPRs, suggesting that these DPRs play a major role in nucleocytoplasmic trafficking defects (Jovičić et al., 2015; Boeynaems et al., 2016; Lee et al., 2016). Although it is possible that GR and PR DPRs indirectly mediate these defects, it is also very likely that the DPRs bind the nuclear pore and directly alter the phase-separation properties of cargoes. Indeed, proteomic analysis revealed that several nuclear pore and trafficking components interact with GR and PR DPRs (Lee et al., 2016; Lin et al., 2016). These DPRs likely interact directly with the core of the nuclear pore, as it has been revealed that the PR DPR can directly interact with and stabilize polymeric phenylalanine: glycine repeats found within multiple nucleoporin proteins, thus changing the dynamic properties of the central channel (Shi et al., 2017).

The relative contribution of GR and PR DPRs to human disease remains an open question. These two DPRs are much less stable than are other RAN-translated DPRs, and the half-life of GR is much shorter than that of PR (Kwon et al., 2014). Future studies will be needed to determine the dose-dependent toxicity of GR and PR DPRs (Kwon et al., 2014; Wen et al., 2014). The *in vivo* translation rate of these DPRs is also unknown, and it is possible that the sense and antisense strands of the hexanucleotide repeat are not translated at comparable rates. It is also likely that the GR and PR DPRs have distinct and separate functions in human disease. Proteomic analysis has shown that ~40% of the GR and PR interactome is shared between these two DPR species (Lee et al., 2016). However, of the total GR and PR interactome, ~35% of proteins bind to only the PR DPR, and ~25% associate with only the GR DPR. There are also distinct differences in the cellular localization of the GR and PR DPRs. The concentration of cytoplasmic GR is greater than that of PR, therefore it is likely that GR plays a greater role in influencing stress granule dynamics (Wen et al., 2014; Lee et al., 2016). Additionally, cytoplasmic GR was found to localize to the mitochondria and associate with mitochondrial ribosomal proteins leading to the induction of oxidative stress (Lopez-Gonzalez et al., 2016). These differences are also reflected in the sub structure of the nucleolus as super resolution imaging has revealed that PR distinctly localizes to the granular component of the nucleolus, whereas GR localizes to both the granular component and the fibrillar core, the central region of the nucleolus that is critical for the production or rRNA (Boisvert et al., 2007; Lee et al., 2016).

Overall, there is strong evidence to suggest that the GR and PR DPRs significantly contribute to the progression of C9orf72-mediated ALS/FTD. The development of mammalian models to study these DPRs is critical to determine the extent to which these DPRs damage neurons and/or glia. Some insights have emerged from a recently described transgenic mouse model in which *C9ORF72* with repeat expansions was expressed using a bacterial artificial chromosome. In this mouse model, motor deficits and neurodegeneration were highly associated with detectable expression of the PR DPR, possibly implicating this species as the most important in disease progression (Liu et al., 2016).

## Role of Other Dipeptide Repeats and the Interaction of Multiple Species

The GP and PA DPRs are uncharged and have a compact flexible coil structure (Figure 2). Consistent with these biophysical properties, these DPRs appear to be relatively inert compared with the other C9orf72 DPR species. In HEK293T and HeLa cells, these DPRs did not appear to alter the subcellular localization of GFP, and mass spectrometry analysis revealed that they do not substantially interact with endogenous cellular proteins (Lee et al., 2016). These species are nontoxic in *Drosophila* models, although the GP DPR localizes to discrete cytoplasmic and nuclear foci in a *Drosophila* model expressing GGGGCC repeats (Mizielinska et al., 2014; Wen et al., 2014; Freibaum et al., 2015; Lee et al., 2016). Additionally, the GP DPR inhibits degradation of a reporter used to assay the functionality of the ubiquitin-proteasome system and increases cell death in the presence of the proteasome inhibitor MG-132 (Yamakawa et al., 2015).

A currently underrepresented area of study in the C9orf72-mediated ALS/FTD field is the interaction of individual DPRs with each other. An initial analysis in Neuro2A cells suggested that GR and PR DPRs recruit both GA and GP DPRs into inclusions (Yamakawa et al., 2015). The interaction between GR and GA DPRs occurs in *Drosophila*, HeLa cells and neurons, and the GR DPR accumulates in cytoplasmic GA DPR containing aggregates. Additionally, expression of the GA DPR mitigates wing abnormalities caused by overexpression of the GR DPR in *Drosophila* (Yang et al., 2015). It is unclear whether the GR DPR affects the sequestration of proteasomal proteins into GA DPR aggregates and whether the PR DPR is also sequestered into GA DPR aggregates. Questions regarding how the five DPR species interact with each other *in vivo* and whether these interactions are mitigating, synergistic, or dynamic (i.e., changing throughout the course of DPR expression and disease progression) remain unanswered.

## Far-Reaching Implications of DPR Production in Degenerative Disease

It is well established that DPRs can induce toxicity in neurons, although the extent to which DPRs play a role in the pathogenesis of C9orf72-mediated disease remains controversial. A summary of what is known about the structures, physico-chemical properties, interactomes and toxic mechanisms of individual DPR species is summarized in Figure 2. It is possible that these toxic mechanisms of DPR expression directly lead to neuronal cell death. However, it is also likely that a number of downstream effectors mediate the process of neuronal death, either directly or indirectly. One likely effector of DPR expression is TDP-43, a major disease protein found in ALS/FTD that was also found to interact with the GR and PR DPRs (Neumann et al., 2006; Lee et al., 2016). Future examination of DPR models, both *in vitro* and *in vivo* will likely yield additional effectors of DPR toxicity.

There are many open questions concerning DPR pathology. It remains to be determined what size DPRs are found in diseased neurons, however it is likely that toxicity is proportional to repeat length as *Drosophila* and yeast models have revealed that toxicity increases as repeat length increases (Mizielinska et al., 2014; Freibaum et al., 2015; Jovičić et al., 2015). Second, it is currently unknown if DPR toxicity is generated in a strictly cell autonomous manner via RAN translation (Mori et al., 2013a,c; Zu et al., 2013). Indeed, a recent publication demonstrated that transmission of all five DPR species can occur through cell-to-cell spreading in neuronal cultures including IPS derived motor neurons. This transmission was found to occur in both the anterograde and retrograde directions via vesicle dependent and independent mechanisms (Westergard et al., 2016). Additionally, it has yet to be determined whether DPR production in glial cells contributes to their toxicity in human disease (Schludi et al., 2015).

An area that requires greater attention is the interplay of DPRs and RNA, specifically with the sense and antisense strands of the hexanucleotide repeats. Of particular interest is the contribution of such interactions to the sequestration of endogenous proteins that are essential for neuronal cell survival. Specifically, both the hexanucleotide repeat RNA and DPRs sequester proteins that are enriched in low-complexity domains and play critical roles in the formation of non-membrane-bound organelles and RNA metabolism (Donnelly et al., 2013; Mori et al., 2013b; Haeusler et al., 2014; Conlon et al., 2016). One example is the sequestration of nucleolin, which associates with both GGGGCC RNA and the GR and PR DPRs (Haeusler et al., 2014). At present, it is unclear if these sequestered proteins associate with both DPRs and hexanucleotide RNA together in a single complex.

Another open question is how the noncoding hexanucleotide sequences undergo translation. It has been proposed that RAN translation is activated by a hairpin structure formed by the repetitive RNA (Cleary and Ranum, 2013). However, it is unclear how this noncoding RNA is exported for translation while avoiding degradation by the nuclear exosome. One hypothesis is that the accumulation of RNA foci may overwhelm the degradation machinery of the exosome, thereby allowing the export of the RNAs for translation. In support of this hypothesis, knockdown of exosomal subunits by RNA interference enhances toxicity in a transgenic C9orf72 *Drosophila* model (Freibaum et al., 2015). Alternatively, or perhaps synergistically, the breakdown of the nuclear pore architecture and defects in nucleocytoplasmic shuttling may lead to leakiness of hexanucleotide repeat RNA from the nucleus into the cytoplasm, where they undergo RAN translation (Freibaum et al., 2015; Jovičić et al., 2015; Zhang et al., 2015, 2016; Boeynaems et al., 2016). Consistent with this hypothesis, cells expressing the expanded hexanucleotide repeat were observed to accumulate excess nuclear RNA (Freibaum et al., 2015; Rossi et al., 2015). This impaired nuclear shuttling may lead to a feed-forward process in which the RAN-generated DPRs are produced with increasingly greater efficiency.

Understanding the interplay between the hexanucleotide repeat RNA and GA, GR and/or PR DPRs will be critical to elucidate the mechanism and temporal process by which neurons die in C9orf72-mediated disease. To summarize what is known about the pathogenic steps that occur during C9orf72-mediated disease progression, we propose the following model: (1) an initial buildup of stable and toxic hexanucleotide G-quadruplex RNA leads to a signaling cascade that leads to the production of RAN DPRs; (2) because the GA DPR is the most stable and abundant species of DPR produced, it most likely plays a primary role in proteasomal inhibition; and (3) finally, the inhibition of the proteasome and further breakdown of nucleocytoplasmic shuttling leads to accumulation of GR and PR DPRs, thereby altering the dynamics of non-membrane-bound organelles and ultimately leading to neuronal death. The association of the GR and PR DPR with a large number of LCDs illuminates how such a vast and disparate array of cellular defects has been observed (see Figure 2). Additionally, it suggests a convergence in mechanisms between DPRs that interact with LCDs leading to alterations in phase dynamics vs. toxicity initiated by disease-causing mutations that directly impact the phase dynamics of specific disease initiating proteins.

## Author Contributions

BDF and JPT co-wrote the manuscript.

## Funding

We are grateful for funding from the Howard Hughes Medial Institute, the ALS Association, the NIH (R35 NS097974) and The Clinical Research in ALS and Related Disorders for Therapeutic Development (CReATe) Consortium (U54 NS092091).

## Conflict of Interest Statement

JPT is a consultant for Inception Biosciences. The other author declares that the research was conducted in the absence of any commercial or financial relationships that could be construed as a potential conflict of interest. The reviewer SR and handling Editor declared their shared affiliation, and the handling Editor states that the process nevertheless met the standards of a fair and objective review.
